# Covid-19 and the Apocalypse: Religious and Secular Perspectives

**DOI:** 10.1007/s10943-020-01100-w

**Published:** 2020-10-30

**Authors:** Simon Dein

**Affiliations:** Queen Mary College, University of London, London, UK

**Keywords:** Apocalypse religious, Secular, Covid

## Abstract

The current Covid-19 pandemic has led to existential crises. One way of finding meaning in this is through apocalyptic narratives. We differentiate between religious (based upon eschatology) and secular apocalypticism (based upon radical political and economic change) and argue that both are to be found in the wake of Covid-19 infection. For religious believers, the apocalypse signifies the rapture of the faithful into heaven while those on earth will undergo the tribulations. For secular believers, the apocalypse signifies sociopolitical change. The paper ends by speculating upon the socio-political and economic changes during and after the pandemic- the New Jerusalem.

Covid-19 is highlighting serious deficiencies in our existing system. An effective response to this is likely to require radical social change. I have argued that it requires a drastic move away from markets and the use of profits as the primary way of organizing an economy (Mair 2020).

Then I saw a new heaven and a new earth, for the first heaven and the first earth had passed away, and there was no longer any sea (Revelations 21:1 KJV).

## Introduction

The coronavirus covid-19 pandemic is the most significant global health crisis of our time and presents the greatest challenge we have faced since the Second World War. To date there have been 9,738,975 cases and 492,390 deaths worldwide as of June 26 2020 (Worldometer 2020). Emerging in Asia in late 2019, the virus is present in every continent except Antarctica. Cases are rising on a daily basis throughout Africa, the Americas, and Europe. While Covid-19 is of course natural, the way it spreads is highly influenced by social, political and cultural factors.

Historically pandemics have been attributed to both religious and naturalistic causes. While epidemics in the ancient world were generally accounted for in religious terms, a concept of contagion still existed (Feder 2013). Throughout history pandemics and apocalyptic narratives have run closely together. As one example explanations of the Black Death (1347–1352) involved ideas of sin and apocalypse, acts of God and alignments of the planets. While bad air, divine punishment and witchcraft were postulated causes, for many the Black Death signaled the end-times (Lerner 1981; Dwyer 2016). In another case, Howard (2008) reports on religious explanations of the 1918 Spanish flu epidemic where appeal was made to ideas of sin and of an eschatological sign of Christ’s second coming. One member of the Dutch Reformed Church, Johanna Brandt, prophesied that the Day of Judgment had come. A pamphlet, *The* *Millennium—A* *Prophetic* *Forecast,* warned readers that the flu epidemic was only the beginning of the affliction that was stated in the Book of Revelations.

Pandemics indicate the fragility of life and the world, chaos, engender paralysing anxiety that the world is dissolving, a sense of detachment and raise significant issues of meaning resulting in existential crises. Self-isolation and quarantine create a sense of being separated from the community and world generally—a sense of anomie. This was summed up well over two thousand years ago by the Roman poet Virgil’s notion of a ‘maze of dread’: ‘The world itself seems entirely unreliable: not only dangerous but also deceptive. The appearance of being a safe and thriving land becomes only a façade that hides the threat of death.’ As Schwetz (2020) states: ‘Pandemics scare us partly because they transform other, less concrete, fears about globalization, cultural change, and community identity into tangible threats.’

And later:

Instead, narratives about contagious disease hold up a mirror to our deepest, most inchoate fears about our present moment and explore different possible responses to those fears.

While the global death rate from Covid-19 has been phenomenal, the virus cannot be divorced from its sociocultural context. But global events are always enacted in a local context and it behoves social scientists to understand the meanings of Covid-19 in specific contexts. As anthropologists would argue, pandemics are more than metrics (numbers, cases, and prevalences), they are embodied, affecting situated lives. Anthropological research is essential for placing the virus in context.

Covid-19 threatens key symbolic frameworks and presents unprecedented challenges for both people and society globally in terms of its impact on mortality, morbidity, economic decline and the ways in which we lead our lives. It has highlighted issues of work, social inequality, globalization, individualism and social interaction generally. Philosopher of science Bruno Latour (2020) discusses the ‘astounding lesson’ that the coronavirus has ‘taught us’: ‘That it is possible, in a few weeks, to put an economic system on hold everywhere in the world and at the same time, a system that we were told it was impossible to slow down or redirect’.

To date much of the scholarly work on religion and Covid-19 has focused on religious practices and the transmission of the virus (Dein et al. 2020). Here I focus upon another aspect of religion-apocalypticism- as one way of understanding Covid-19. It is religious fundamentalists who generally associate coronavirus as a sign of end times or a final judgment. But apocalypses can be secular as well as religious. In the time of Covid-19 it has become obvious that the current world order is becoming a thing of the past and the future is highly likely to be different.

Peter Berger (1967) described a ‘plausibility structure’ as the ‘symbolic base’ that every society ‘has to continuously construct and maintain for assuring its existence as a world’. Our current plausibility structures upon which the existence of society is dependent and threatened and we are urgently in need of alternative sociocultural contexts to provide structures of meaning.

## Religious Apocalypticism

While in the lay mind the term apocalypse signifies some violent and cataclysmic ending of the world, the word derives from *Apokalypsis*—the Greek word for Revelation. Aldrovandi (2014) discusses the etymology of this word:

Apokalypsis’ in the original, etymological understanding of the word: a sudden breaking point in human destiny unveiling an ultimate truth (*aleitheia*) that has always been present, but remains most of the time hidden, denied or forgotten.

The idea was brought down from the Book of Revelation, the final text of the Christian Bible, and denotes a time when some truth or understanding, previously unknown, is revealed. Apocalyptic literature, as a genre, documents the authors' visions of the end times which have been revealed by an angel or some other heavenly messenger.

Fundamentalist Christians, especially those who hold to the rapture, assert that the Book of Revelation forecasted the pandemic 2000 years ago. Throughout the course Christian history, apocalyptic expectations that the world will imminently end have waxed and waned. The idea that history is moving inexorably to a catastrophic end is a longstanding one in the West. The millennial years, 1000 and 2000 were associated with heightened apocalyptic expectations. Catastrophic events, such as the Black Death in 1348 or the Cuban Missile Crisis in 1962, regularly bring about panic and the expectation that doomsday is imminent. History is replete with groups maintaining that the end of the world is imminent: Millerites, Shakers, Jehovah’s Witnesses, members of the Oneida Community, Mormons, and Seventh Day Adventists, Rappites, Christadelphians, and Native American millennialists to name just a few.

Apocalyptic narratives, particularly premillennialist, have significantly impacted American religion and politics and fuelled evangelical political activism and continue to exert a great influence over the contemporary American mainstream (Sutton 2014). Similarly, Kyle (2012) notes how dispensational premillennialism drives doomsday ideas in the USA. For this author apocalyptic thought “breaks out” and on occasion “infects the wider culture” (xi–xii). Pre-millennialism asserts that the Second Coming of Christ is imminent, that Biblical prophecies provide clues as to when it will take place, and that when it occurs all true believers will be ‘raptured’ to heaven while everyone remaining on Earth will endure the ‘tribulation’. For this author radical Evangelical Christianity is inextricably linked to free market economics. Those who maintain the immanence of the apocalypse continuously attempt to align biblical prophecy with contemporary world events.

Until the beginning of the twentieth Century apocalyptic narratives were generally the purview of religion. According to Kyle (2012) the durability of Apocalyptic ideas in the face of continual disconfirmation results from the consolations they provide in the wake of global adversity. However, it is not just fundamentalist Christians who associate adverse events with the end of the world. Weber (2004: 11) notes:

More than one-third of Americans said that since the terrorist attacks of 9/11, they have been thinking more about how current events might be leading to the end of the world. While only 36 percent of all Americans believe that the Bible is God’s Word and should be taken literally, 59 percent say they believe that events predicted in the Book of Revelation will come to pass. Almost one out of four Americans believes that 9/11 was predicted in the Bible, events predicted in the Book of Revelation [in particular, the battle of Armageddon] will come to pass. and nearly one in five believes that he or she will live long enough to see the end of the world.

Some groups actively prepare for the apocalypse. Garrett (2020) reports on USA preppers—individuals who prepare for the imminent cataclysm by hiding in underground bunkers. The term prepping according to Garrett refers to practices of anticipating and adapting to impending conditions of calamity. These range from low-level crises to ‘extinction-level events’.

Since it was first written in 95 AD by John of Patmos, the Book of Revelation has deployed to account for world events in terms of the ‘end times’. It was finally accepted as the last book of the Christian Cannon in 367CE by Bishop Athanasius of Alexandria. While there is disagreement among scholars as to how the text is to be interpreted, the book spans three genres: Epistolary, the apocalyptic, and the prophetic. Many have taken the text as a literal description of the end times while others have used it as a revelation of divine will. The book provides meaning to the past and future by viewing all events as a part of god’s plan. Yet others see it as a social allegory-a diatribe against the oppression of imperial Rome. And for others it is a direct criticism of power concentrated in the hands of a small elite and systemic injustice both resulting in poverty, famine, hunger, pestilence and disease. In terms of Revelations, the Beast from the Sea (Chap 13) and the woman (Chap 17) represent the Roman Empire (Collins 1977) and represent a political perspective in this book. Reidl (2014) notes that the apocalypse is a millennia-old symbolic complex which emerged in Jewish intellectual circles who were experiencing oppression and social and political alienation as a result of foreign imperial power. For him apocalypses are characterized by a complete rejection of present reality and an outlook toward an entirely different future reality where power relations will be reversed.

Pagels (2012) argues that apocalyptic literature which includes visions, prophecies and predictions of cataclysm always carries political overtones, both revolutionary and reactionary, liberal and conservative. This is the case from its earliest beginnings until today, as can be found in conservative iterations of millennial dispensationalism and the highly popular ‘Left Behind’ series of novels concerning the world’s ending. In her view apocalyptic meanings are always malleable and can adapt to any crisis. I would argue that its use as political critique is one reason for the widespread appeal of the book throughout the last two thousand years or so.

Revelation describes four horseman of the Apocalypse who appear when the seven seals are opened. The first symbolizes Christ. The second represents war and bloodshed. The third is identified with famine and the fourth is associated with pestilence and death. Some Christians claim that COVID-19 is proof that the plagues of the book of Revelation, and in particular the seven Seals of Revelation 6:1–8:1, are occurring now and Jesus’ return is imminent. For them, Revelation has indeed predicted the COVID-19 pandemic. Corona has been associated with the fourth horseman.

Lindsey and Carson’s (1970) The *Late* *Great* *Planet* *Earth* asserted a close relationship between end-of-time prophecy with then-current events and soon became the most popular non-fiction book of the 1970s, with over 28 million copies sold by 1990. Its author claimed that the rapture would occur by 1988. This book, alongside numerous other films and television series since, resulted in a transition of apocalyptic thinking from fringe religious belief to fundamental tropes which are popular and known to many in contemporary American culture.

Finally, the Left Behind series of 16 bestselling religious novels by Tim LaHaye and Jerry B. Jenkins, deals with Christian dispensationalist interpretation of prophecies in the Biblical books of Revelation, Daniel, Isaiah and Ezekiel, in which true believers in Christ have been "raptured", leaving the world in a chaotic state. These books dating from 2000 have had a profound influence on apocalyptic though in the USA (Forbes and Halgren Kilde 2004).

Christian social media threads like #Jesusiscoming are replete with discussions of the imminence of the end times and *Watch* *Jerusalem* editor-in-chief and Evangelist pastor, Gerald Flurry (2020), recently asserted the coronavirus is a sign from God redirecting humanity on the right path before the ultimate clash between the forces of good and evil:

God is pleading, he’s pleading. We know there’s all kinds of imminent pestilence epidemics that are going to sweep across this world during the Great Tribulation. The epidemic, disguised as one of the Four Horsemen of the Apocalypse, will wipe out a quarter of the planet’s population.

In recent months, Christian sources on the internet quote a range of biblical sources about the preceding of the end of time with pestilence. There are several examples: Revelation 6:8—And I looked, and behold a pale horse: and his name that sat on him was Death, and Hell followed with him. And power was given unto them over the fourth part of the earth, to kill with sword, and with hunger, and with death, and with the beasts of the earth. Matthew 24:7–13—For nation shall rise against nation, and kingdom against kingdom: and there shall be famines, and pestilences, and earthquakes, in diverse places. Ezekiel 38:22—“I will plead against him with pestilence and the blood.”

But Revelation is not the only book in the Christian Bible to speak about an end-time return of disease outbreaks. According to the Gospels, Jesus himself stated that pandemics of pestilence would happen before His return: “For nation shall rise against nation, and kingdom against kingdom: and there shall be … pestilences … in divers places” (Matthew 24:7; see also Mark 13:8 and Luke 21:11). Pestilence in this context refers to full scale disease outbreaks.

### Secular Apocalypticism

Unlike religious apocalypticism where the future is determined by divine intervention, in secular apocalypticism natural events such as the current Covid-19 pandemic are the cause of the impending doomsday. Kyle (2012) argues how secular apocalypticism has grown out of a ‘naturalistic’ world view and has frequently involved environmental factors like climate and nuclear destruction. Apocalyptic belief was invigorated by the World Wars, the Cold War, and the threat of nuclear annihilation. Fears pertaining to atomic destruction, lethal disease, and environmental catastrophes have become prevalent. Specifically, I argue that the Covid-19 pandemic has been viewed in apocalyptic terms signifying a radical societal change.

This author notes how the secular and religious apocalypticism are mirror images of each other. Both originate from disasters, social dislocation and dashed expectations. He asserts that the early secular apocalypse grew out of its religious counterpart and until the beginning of the twentieth century there were significant interactions between the two. Even now the idea that God deploys the natural world (e.g. tsunamis) or technology (e.g. nuclear power) to punish humanity remains contentious among religious people; the natural, divine and human often provide overlapping explanatory models of misfortune. By no means new, notions of a secular apocalypse have featured highly in Karl Marx’s end of capitalism and Francis Fukuyama’s end of history.

Both secular and religious forms offer hope for change. As Shermer (2011) notes:

Emotionally, the end of the world is actually a renewal, a transition to a new beginning and a better life to come. In religious narratives, God smites sinners and resurrects the virtuous. For secularists, the sins of humanity are atoned through a change in our political, economic or ideological system.

Stephen O’Leary (1994) notes that the latter half of the twentieth century brought with it a ‘resurgence of popularity’ in apocalyptic thought due to the threat of nuclear war, which made religious forecasts of ‘planetary destruction credible to a much wider audience’. Apocalypse has now become a popular cultural trope in fiction, film and popular discourse. Far from dissipating in modernity, apocalypticism appears to be here to stay and is possibly escalating. Originally deriving from the millennial fervour of the English Puritans coming to the USA in the seventeenth Century and the utopian hope of developing a ‘New Jerusalem’ on American soil, Kyle (2012) asserts that apocalyptic themes have reached near ‘epic proportions’ in contemporary American society. Like religious apocalyptic thinking, the secular version is one response to the problem of suffering- in theological terms a theodicy.

In modernity science is the primary lens through which humans understand their worlds. While science provides hope for a better world through technological innovation, the possibility of a man made apocalypse becomes ever more probable and apocalyptic narratives move from hope and meaning to the real possibility of mankind’s self-destruction.

Akin to the notion of secular apocalypse is the term Cultural Apocalypse—a term first coined by Italian anthropologist Ernesto De Martino (1964) to describe the sense that the current historical epoch is coming to an end. De Martino characterizes such apocalyptic experience as follows:

Experiencing an apocalypse implies first of all a “loss of presence”, that is, being cast outside any possible secular or religious horizon of salvation, completely detached from the familiar, facing without any comfort the diabolical unhinging of all that has been known.

Psychoanalyst Robert Lifton (1979) deploys the term ‘symbolic death’ to denote the dissolution of society, something he notes can be more frightening than biological death. Both De Martino and Lifton assert that traumatic breakdown has a regenerative side, offering hope that society will continue in some (radically changed) form and provide not so much an ending, but rather, a new beginning. And O’Leary (1994) argues that apocalyptic scenarios have always been of interest since they provide a sense of meaning to existence, providing purpose and direction to the future. Not only do they enable understanding of the present but they also provide hope for the future.

Aldrovandi (2014: 202) notes how apocalyptic ideas facilitate coping with:

absurdity and despair, especially in trying times. It has been repeatedly argued that the apocalyptic is a ‘self- contained’ myth whose strength resides in a dialectic binding together of dissolution and renewal’. Seeing the world through an apocalyptic prism entails the possibility that even the most unsettling catastrophe can be symbolically tamed by being interpreted as a sign that a phase of deficiency is reaching its nadir and a new beginning is at hand. In a similar vein Griffin (1991) asserts how apocalyptic myth provides an opportunity for the most remarkable chance of self- renewal.

Thus all apocalyptic narratives deploy the plot device of the end of the world to examine contemporary society and facilitate exploration of alternative social structures.

Yar (2020) compares religious and secular forms of apocalypticism, Both are utopian and view something positive as coming from adversity:

After all, the origins of such discourse are rooted firmly in religious anticipations of redemption – the end times mark the realization of justice deferred, an end to suffering and exile, restitution for the sufferings inflicted upon the innocent, and reconciliation of humans with each other, nature, and/or the divine. In other words, the horrors of all-encompassing disaster simultaneously contain the seeds of utopia. In its modern, secularized form, this ambivalence manifests in a hope of social renewal, the idea that out of crisis the supposed alienations of modernity can be countered by a rediscovery of solidarity and community.

It is in this context that we might situate, for example, the words of António Guterres, Secretary General of the United Nations, when he asserts that “recovery from the coronavirus crisis must lead to a better world”. Whether or not the current “rediscovery of the social” will in fact endure beyond the horizon of the crisis is a matter of conjecture, but it seems clear to me that the imaginaries of disaster, suffering, hope and renewal that configure apocalyptic culture are alive and well as we navigate the challenges of our present straits.

So how does Covid-19 relate to Apocalyptic thinking? As Stein (2020) states’ It's not surprising that the pandemic surrounding us has been compared to the apocalypse, as people are trying to find a meaning in this plague.’ Schut (2013) reminds us how apocalyptic narratives include a distinct element of ‘after the end’ leading to a post-apocalyptic world. This author underscores the fact that the apocalypse provides a critique of the philosophy and structure of consumption-based capitalism, undercuts its old ways of thinking and living and reveals the weaknesses of these structures which prove difficult to see from within.

### Covid-19, Revelation, Politics the New Jerusalem

What has Corona revealed and what might be the social and economic implications of it? The pandemic pushes individuals to reflect on the current social status quo. Old assumptions and ways of life are questioned leading to a new beginning. Like the Book of Revelation, contemporary apocalyptic discourse is a critique of unequal power relationships and the uneven distribution of wealth in capitalism. As Goldberg (2020) points out, the Covid-19 crisis has given rise existential debates about how we should think about the ‘after-Covid world’.

The Covid crisis is revealing health care inequalities, class divisions, unequal distribution of power, and the fact that the most important workers in American society are among the least paid. Health inequalities have been brought into sharp focus and the crisis has exposed the structural disadvantage and discrimination faced by parts of the black, Asian and minority ethnic communities. Our taken for granted ’natural’ ways of behaving are increasingly being challenged. As Davis (2020), a sociologist and political economist, writes:

‘It will take years or decades for the significance of 2020 to be fully understood. But we can be sure that, as an authentically global crisis, it is also a global turning point. There is a great deal of emotional, physical and financial pain in the immediate future. But a crisis of this scale will never be truly resolved until many of the fundamentals of our social and economic life have been remade.”

While the sudden end of ways of life has been a regular event throughout history, Covid-19 has changed the ways we live and interact in ways previously unimaginable to us. Perhaps the effects of the pandemic can be better compared to the effects of terrorism, albeit the numbers of people killed is much smaller, with its increased surveillance of populations by security cameras and security procedures, rather than to previous epidemics like the Spanish flu of 1918 which did not give rise to major social change subsequently. Now social distancing is carefully monitored and freely intermingling appears to be a thing of the past. In Foucault’s terms, new forms of bio power have arisen. Our lives have become more physically restrained. But at the same time we are reconnecting with nature, taking long walks during the lockdown.

Some have predicted how the pandemic will be a once in a generation opportunity to remake society and provide a better future. While no one can be sure how the novel coronavirus will change the world we live in, it is likely that the near future will dramatically different from the one we knew pre-covid-19 in terms of the ways we live, work and worship. Covid-19 has forced many countries to reconsider their social policies, particularly social protection and healthcare. There has been a move from profit to protection of life. Attempts to help workers in the informal sector could potentially diminish inequality. Those workers who have been undervalued and underpaid before the crisis, such as healthcare and key workers may gain prestige and power, resulting in better quality jobs after it. There may well be more opportunities to address systematic barriers facing black, Asian and other ethnic minority groups.

In terms of economy, some have claimed the end of capitalism as we know it with wages, labour and consumption being influenced by the pandemic and questions are being raised about the validity of our assumptions about markets (Mair 2020). This philosopher and staunch critic of globalization argues: ‘Liberal capitalism is bust.’

One issue that arises from the pandemic is what is an economy for? Is it to buy, sell and make or profit-to facilitate the exchange of money, or rather should it function to provide the things we need to live? Mair (2020) asserts: ‘Covid-19 is highlighting serious deficiencies in our existing system. An effective response to this is likely to require radical social change. I have argued that it requires a drastic move away from markets and the use of profits as the primary way of organizing an economy.’ In times of Covid-19 governments have stepped in and are injecting money into the economy as opposed to businesses steer and create wealth. Mazzucato (2020) underscores the fact that the pandemic reveals a lack of preparedness and resilience of the increasingly interconnected global economy.

Finally some analysts maintain that all services will become digitalized. In particular, health service provision is likely to feature digital technology with telemedicine becoming more prominent and more reliance on home testing. This may well extend to religious worship where, for the first time in history, religion has come online and real-life worship has been put on hold.

## Conclusion

This paper has examined the parallels between religious and secular apocalypticism. Both have been found in the wake of the Covid-19 pandemic. Both are direct responses to the existential crisis engendered by the virus and underlying inequalities in power structures. What societal changes will occur post Covid and how long they endure remains to be seen.
